# Diode Laser Irradiation for Cavity Decontamination: A Pilot In Vitro Study on Antimicrobial Efficacy

**DOI:** 10.4317/jced.64128

**Published:** 2026-05-29

**Authors:** Francesca Zotti, Giacomo Piersilvio Zocca, Giorgia Lanzaretti, Caterina Signoretto

**Affiliations:** 1 Department of Surgical Sciences, Pediatrics and Gynecology, University of Verona, P.le L.A.Scuro, 10, 37134 Verona, Italy; 2 Private Practice Vicenza, 36030, Italy; 3 Department of Diagnostics and Public Health, University of Verona, P.le L.A.Scuro, 10, 37134 Verona, Italy

## Abstract

**Background:**

This pilot study evaluated the potential of a 980 nm diode laser as a viable option for dental cavity decontamination, comparing its antimicrobial effect with 0.2% chlorhexidine.

**Material and Methods:**

Forty-seven extracted human molars with standardized Class I preparations were sterilized, conditioned with sterile stimulated saliva, and inoculated with Streptococcus mutans. The teeth were arbitrarily assigned to three groups: no treatment (n = 7), chlorhexidine 0.2% for 30 seconds (n = 10), and irradiation with a 980 nm diode laser for 30 seconds (n = 30). After incubation in BHI broth for 24 hours at 37°C, bacterial growth was quantified as CFU/mL using non-parametric tests.

**Results:**

Both treatments produced a marked reduction in bacterial growth compared with the untreated group (p &lt; 0.001). Chlorhexidine completely suppressed detectable growth, whereas the laser showed residual contamination in a few samples, though always at much lower levels than the control. The difference between chlorhexidine and laser was not statistically significant (p = 0.253).

**Conclusions:**

Despite its small scale, this pilot study indicates that 980 nm diode irradiation can substantially lower S. mutans levels in contaminated cavities. While not conceived as a substitute for chemical disinfectants, the laser presents characteristics that merit attention: no chemical residues, no contribution to bacterial resistance, and potential implications for future adhesive-interface investigations. These aspects may justify further investigation into its use as an adjunctive step within restorative procedures, particularly in protocols aiming to limit chemical agents or to enhance substrate conditioning.

## Introduction

The use of laser technology in dentistry has progressively evolved from an experimental adjunct to a clinically relevant tool across several disciplines. Among the available devices, diode lasers have gained particular attention because of their compact design, ease of handling, and favourable absorption in pigments and bacterial chromophores (1). Their applications span soft-tissue management, periodontal decontamination, and photobiomodulation, yet their role within restorative dentistry remains less clearly defined. Although they are not indispensable in routine restorative practice, certain characteristics of diode lasers make them worth exploring in the context of cavity disinfection. Residual bacterial contamination within a prepared cavity is a well-acknowledged risk factor for restorative failure and post-restorative sensitivity, particularly when Streptococcus mutans persists at the adhesive interface (2 - 3). Mechanical excavation alone does not always ensure complete microbial removal, and viable microorganisms can survive even in apparently sound dentin (4). For this reason, chemical disinfectants, most notably chlorhexidine, are commonly incorporated into adhesive protocols to reduce microbial load before bonding procedures. Despite its established efficacy, chlorhexidine presents several drawbacks, including potential cytotoxicity, concerns regarding its long-term effect on dentin bonding, and an increasing tendency toward more selective and conservative use of chemical agents in restorative workflows (5 - 6). Furthermore, studies indicate, in particular in long-term application, a growing resistance to chlorhexidine in bacterial oral pathogens, including S. mutans (7). These considerations have stimulated interest in laser-based decontamination as a non-chemical alternative. The antimicrobial effect of diode lasers relies primarily on thermal disruption of bacterial structures, which does not contribute to the development of antimicrobial resistance and does not leave chemical residues on the dentin surface (8 - 10). Moreover, preliminary investigations have suggested that laser irradiation may induce morphological changes in dentin that could influence adhesive performance (11 - 13) an aspect that, although not yet conclusively demonstrated, justifies further investigation. At the same time, routine use of lasers for disinfection cannot be assumed as clinically justified: devices are costly, technique-sensitive, and their added value must be supported by evidence rather than technological appeal. Given these premises, the present work was designed as a pilot study, intentionally limited in scale, to gain preliminary insight into whether a 980 nm diode laser can meaningfully reduce S. mutans contamination inside standardized Class I cavities. The novelty of the present study does not reside in the concept of laser-assisted cavity decontamination itself, which has already been explored in the literature, but rather in the standardized evaluation of a specific 980 nm diode laser irradiation protocol under controlled operative conditions. The comparison with 0.2% chlorhexidine-currently among the most widely adopted cavity disinfectants-provides a practical benchmark to contextualize the laser's performance. The aim of this study was to quantify the reduction of viable S. mutans (expressed as CFU/mL) after laser irradiation or chlorhexidine application, compared with an untreated control group. By measuring both the presence and the magnitude of residual bacterial growth, this investigation aims to clarify whether diode irradiation warrants further exploration as an adjunctive strategy within restorative procedures, particularly for clinicians seeking alternatives that limit chemical exposure and avoid contributing to antimicrobial resistance. Based on the known photothermal antibacterial effects of diode lasers, the null hypothesis (H0) of this study was that 980 nm diode laser irradiation would not produce a significant reduction in viable S. mutans counts compared with untreated cavities, nor differ from 0.2% chlorhexidine disinfection. The alternative hypothesis (H1) was that laser irradiation would significantly reduce bacterial load and show an antimicrobial effect comparable to chlorhexidine.

## Material and Methods

**Figure 1:**
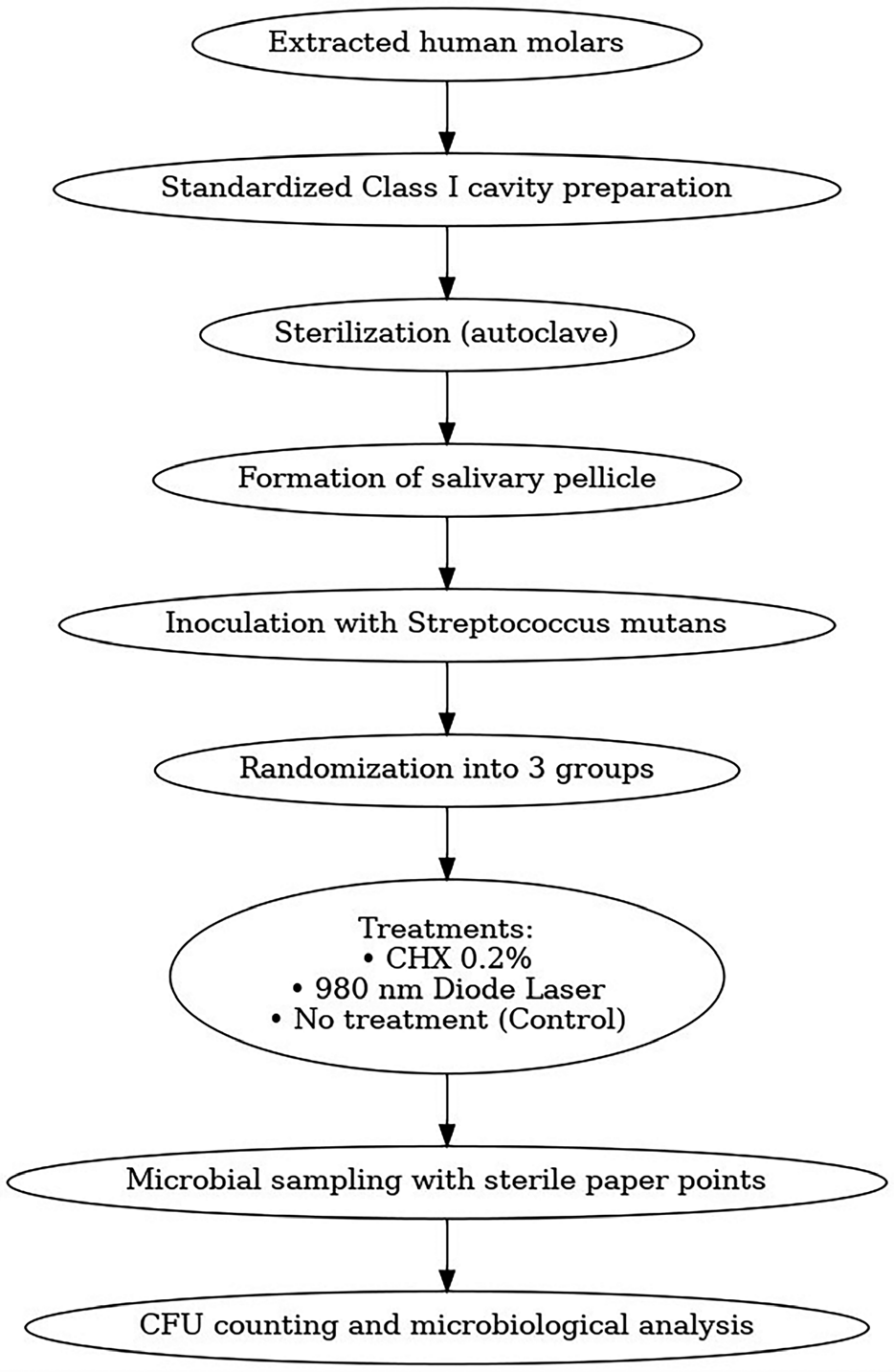
Schematic representation of the experimental workflow. Extracted human molars underwent standardized Class I cavity preparation and sterilization, followed by salivary pellicle formation and Streptococcus mutans inoculation. Specimens were randomized into three groups (chlorhexidine, diode laser, control), treated accordingly, sampled using sterile paper points, and analyzed by CFU counting.

- Study Design and Sample Allocation This pilot study was conducted in accordance with established methodological guidance for pilot and feasibility research, including the CONSORT extension for pilot studies and WHO/TDR recommendations, which permit the use of smaller and occasionally unbalanced groups when the aim is exploratory rather than confirmatory (14). A total of 47 extracted human molars were included and assigned to three groups: untreated control Group (C): (n = 7), chlorhexidine 0.2% named Chlorhexidine Group (CHX) (n = 10), and diode laser irradiation named Laser Group (L) (n = 30). The unbalanced distribution was intentional, allowing a broader assessment of variability within the laser-treated specimens, which represented the primary focus of the study. Randomization was performed using a simple allocation sequence generated in Excel (Excel version 16.84, Microsoft Office, 2024), with no stratification. All procedures were approved by the South-East Veneto Ethics Committee (430CET). A schematic representation of the experimental workflow is presented in Figure 1.

1

- Selection and Preparation of Teeth Forty-seven sound human molars, free from caries, cracks, or restorations, were collected and cleaned with an ultrasonic scaler. Teeth were stored in sterile saline solution until use. Standardized Class I cavities (5 mm depth; 4 mm mesiodistal width; 4 mm buccolingual width) were prepared on the occlusal surface using diamond burs under water cooling. After preparation, the cavities were rinsed with distilled water, and all specimens were sterilized in an autoclave at 121 °C for 15 minutes. Sterile teeth were stored in sealed sterile bags at room temperature until the experimental phase. - Preparation of Stimulated Saliva Stimulated saliva was collected from healthy adult donors with no recent antibiotic or antimicrobial mouthwash use. Donors chewed paraffin wax for five minutes, after which the saliva was centrifuged at 14,000 rpm for 30 minutes at 4 °C. The supernatant was filtered through a 0.22 µm sterile membrane. To confirm sterility, 200 µL of the processed saliva were plated on Schedler agar (Liofilchem® S.R.L., Italy) and incubated anaerobically for 72 hours at 37 °C. Only sterile batches were used. Aliquots were stored at -20 °C and thawed immediately before use. - Bacterial Strain and Inoculum Preparation Streptococcus mutans ATCC 25175 was cultured in Brain Heart Infusion (BHI) broth (Liofilchem® S.R.L., Italy) and incubated at 37 °C in 5% CO2 for 24 hours. The bacterial suspension was adjusted spectrophotometrically to approximately 5 × 106 CFU/mL, and used as the inoculum for dental cavity contamination. - Contamination Protocol Each sterilized cavity was inoculated with 150-200 µL of sterile stimulated saliva and incubated in aerobic atmosphere for 24 hours at 37 °C to allow the formation of a conditioning film. Subsequently, 150-200 µL of the S. mutans inoculum were placed inside each dental cavity, followed by a second 24-hour incubation at 37 °C in 5% CO2. This two-step protocol allowed bacterial adhesion and the early formation of a biofilm-like layer, simulating a contaminated cavity prior to restorative procedures. - Treatment Procedures 1. Control Group (C): Bacterial inoculation only, no disinfecting treatment was performed. 2. Chlorhexidine Group (CHX): Bacterial inoculation then a 0.2% chlorhexidine gluconate solution (Broxo® Din Chlorhexidine, SIT Laboratorio Farmaceutico SRL, Italy) was applied with a sterile dropper for 30 seconds. Excess solution was aspirated, and the cavity was gently air-dried under sterile conditions. The 30-second application time was chosen based on widely adopted restorative protocols (5). 3. Laser Group (L): Bacterial inoculation then a laser irradiation was performed using a 980 nm diode laser (Doctor Smile Wiser 3, Lambda SpA, Italy) in continuous-wave mode at 2.5 W assured by the machine setting known as "decontamination mode". A 320 µm fiber tip was used in non-contact mode, positioned approximately 1 mm above the cavity floor. Before irradiation, cavities were gently dried with sterile paper points to maintain a moist but not wet substrate, minimizing uncontrolled thermal rise. Each cavity was irradiated for 30 seconds using slow circular scanning movements to ensure homogeneous exposure of all internal surfaces of the wet cavity. The 30-second circular irradiation time was selected to mirror the duration of the chlorhexidine application and to maintain comparability between groups. - Sampling of Treated Cavities After treatment, microbial sampling was performed using sterile endodontic paper points (Autfit, Kerr Endodontics). Each point was inserted into the cavity for 30 seconds and then transferred into an Eppendorf tube containing 150 µL of sterile saline. The tube was vortexed for 60 seconds to release bacteria into the solution. - Serial Dilutions and Plating Serial ten-fold dilutions (100 to 10-3) were prepared in sterile Phosphate Buffered Saline (PBS) using a 96-well microplate. From each dilution, 20 µL were transferred to BHI agar plates using the dropwise dilution technique, ensuring triplicate drops per dilution. The plates were incubated at 37 °C in 5% CO2 for 24 hours. This method allowed quantification of viable colonies while minimizing plating variability. - Colony Counting and Data Transformation After incubation, colony counting was performed using a digital colony counter and expressed as Colony Forming Units per ml (CFU/mL). For statistical analysis, CFU/mL values were converted to logarithmic scale (log10 CFU/mL) to improve interpretability while preserving the non-parametric approach required by the dataset. - Statistical Analysis Given the skewed distribution of CFU/mL values and the high frequency of zero counts in treated groups, non-parametric tests were adopted. The Kruskal-Wallis test assessed differences among the three groups, followed by Bonferroni-adjusted pairwise comparisons. The presence/absence of bacterial growth was evaluated using Fisher's exact test. Analyses were performed using Stata software (17.0 version, StataCorp LLC, College Station, TX, USA), with statistical significance set at p &lt; 0.05.

## Results

A non-parametric analysis was chosen because CFU/mL values were highly skewed, and many treated samples showed zero growth. The Kruskal-Wallis test identified a significant overall difference among the three groups (p &lt; 0.001), and this pattern was confirmed when data were examined both as absolute CFU/mL and as log10-transformed values. - Bacterial Growth Among Groups All specimens in the untreated control group showed substantial bacterial growth (Table 1), with CFU/mL values ranging from 1.55 × 106 to 6.50 × 106 and a median of 2.30 × 106 CFU/mL.

Table 1

In contrast, none of the specimens treated with 0.2% chlorhexidine showed detectable colony growth. The diode-laser group presented a mixed pattern: most samples showed no growth on the plate, while a small number (5 out of 30; 16.7%) showed residual growth. In these latter, bacterial growth levels were markedly lower than those of the control group, with values ranging from 3.00 × 102 to 2.84 × 104 CFU/mL. - Comparisons Between Groups Pairwise comparisons with Bonferroni correction showed significant differences, as presented in Table 2.

Table 2

These results indicate that both treatments were effective at reducing viable S. mutans, although chlorhexidine achieved complete suppression. Binary Growth Analysis When bacterial growth was evaluated as a binary variable (presence/absence), Fisher's exact test confirmed substantial differences among groups (Table 3): Control: 7/7 samples positive (100%) Chlorhexidine: 0/10 positive (0%) Laser: 5/30 positive (16.7%) This pattern aligns with the quantitative results and supports the antimicrobial effect of both treatments.

Table 3

**Figure 2:**
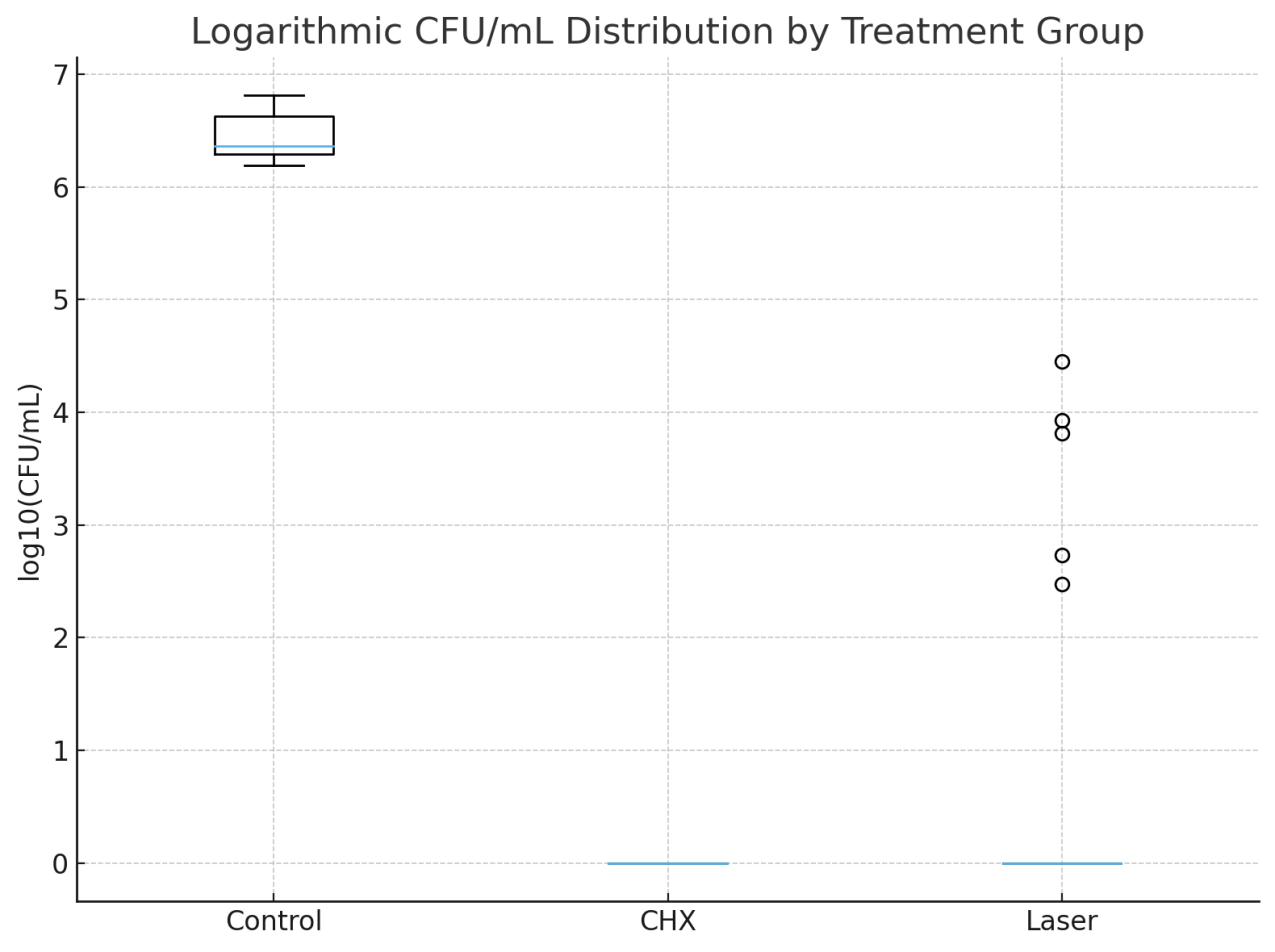
Boxplot with CFU/ml logarithmic values.

- Logarithmic CFU/mL Values Among samples that did exhibit growth, the median log10(CFU/mL) was 6.36 in the control group and 3.81 in the laser group, confirming a markedly lower microbial load following laser treatment. Graphical representation with boxplots further illustrated the wide separation between the control specimens and both treated groups, particularly after logarithmic transformation (Fig. 2).

2

**Figure 3:**
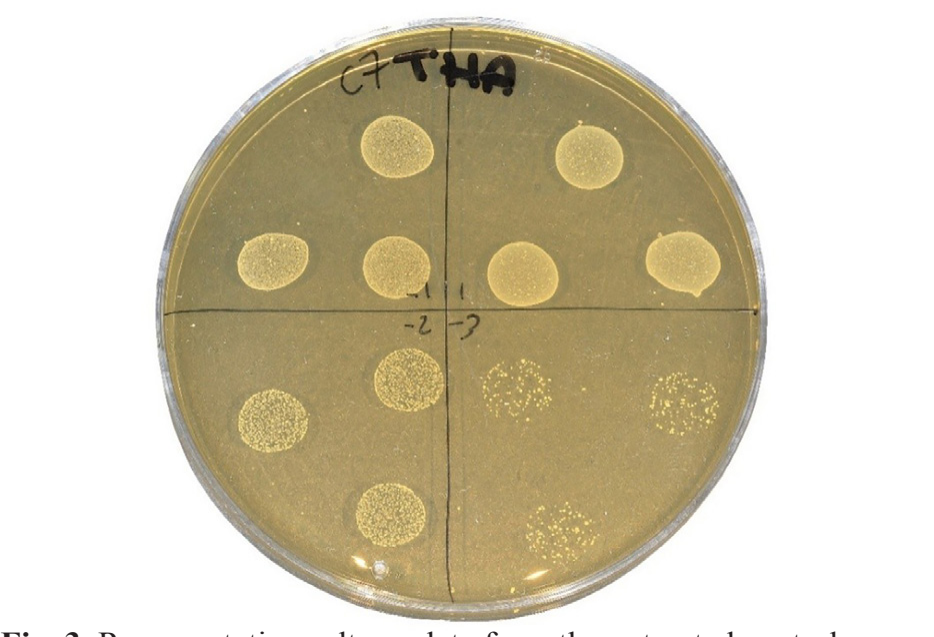
Representative culture plate from the untreated control group. Bacterial growth is visible on the culture medium plate at all dilutions. The numbers on the plates (1,-1,-2,-3) indicate the dilution factor.

**Figure 4:**
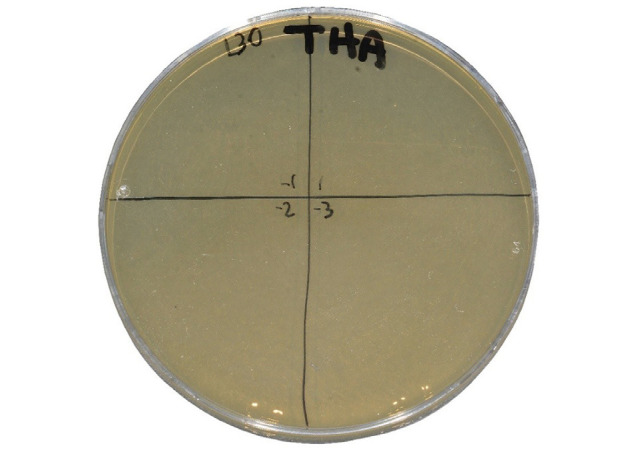
Representative culture plate from the laser group. No bacterial growth is visible on the culture medium plate.

**Figure 5:**
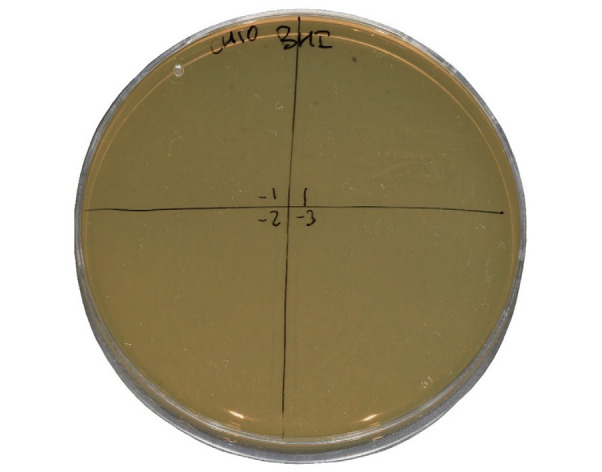
Representative culture plate from the chlorhexidine group. No bacterial growth is visible on the culture medium plate.

Several images showing representative culture medium plates have been included (Figs. 3,4,5).

3

4

As this investigation was conceived as an exploratory pilot study, the sample size was not determined using formal power calculations. Pilot and feasibility guidelines (WHO/TDR and CONSORT) recommend that sample size in early-stage laboratory studies be guided primarily by the exploratory purpose of the study, allowing for smaller and occasionally unbalanced groups when the aim is to assess methodological feasibility and obtain preliminary effect estimates. This rationale guided the decision to include a larger number of laser-treated samples, given that the primary objective was to characterize the variability and magnitude of response associated with diode irradiation, rather than to conduct a fully powered comparative trial.

## Discussion

This pilot study explored the antimicrobial potential of a 980 nm diode laser in the decontamination of standardized Class I dental cavities, using 0.2% chlorhexidine as a benchmark treatment. The findings of this preliminary study indicate that both 0.2% chlorhexidine and 980 nm diode laser irradiation were able to reduce S. mutans contamination within standardized Class I cavities, with chlorhexidine achieving complete suppression under the conditions tested. Although the laser treatment did not completely eliminate bacterial growth in all samples, the residual counts were markedly lower than those of the untreated control group, suggesting a meaningful antimicrobial effect that aligns with previous reports on the bactericidal capabilities of diode lasers through photothermal disruption of bacterial cell structures (15 - 16). In particular, the difference observed between the untreated control group and the laser-treated group reached statistical significance, confirming that diode irradiation produced a measurable reduction in bacterial load compared with no treatment. As this investigation was conceived as an exploratory pilot study, the sample size was not determined using formal power calculations. Pilot and feasibility guidelines (WHO/TDR and CONSORT) recommend that sample size in early-stage laboratory studies be guided primarily by the exploratory purpose of the study, allowing for smaller and occasionally unbalanced groups when the aim is to assess methodological feasibility and obtain preliminary effect estimate (14). This rationale guided the decision to include a larger number of laser-treated samples, given that the primary objective was to characterize the variability and magnitude of response associated with diode irradiation, rather than to conduct a fully powered comparative trial. Several methodological considerations should be acknowledged. On the one hand, the study relied on an in-vitro model using extracted molars, which, despite allowing a high level of standardization, cannot fully replicate the complexity of the oral environment. On the other hand, the contamination model was intentionally limited to a single bacterial species, S. mutans. Although this organism is a central actor in the initiation of dental caries, it represents only one component of the polymicrobial cariogenic biofilm (17). Established multispecies models, including combinations of Lactobacillus spp., Actinomyces spp., and Veillonella spp., more accurately mimic in vivo biofilm ecology, and future work should expand toward such systems to determine whether diode irradiation exerts a comparable effect in a more realistic microbial setting. Another point deserving attention concerns the interaction between surface conditioning and adhesion. A growing body of literature suggests that diode irradiation may alter dentin morphology, particularly through smear layer modification and changes in surface energy, and may influence the penetration and polymerization behavior of adhesive systems. Although these aspects were not assessed in the present study, they represent a potentially valuable area of investigation, especially given the ongoing interest in optimizing the adhesive interface to improve long-term restoration stability (18). From a clinical perspective, the comparison with chlorhexidine is relevant, as it is considered the gold standard and is the most commonly used antimicrobial agent for cavity disinfection. However, its limitations are increasingly recognized, including concerns regarding cytotoxicity, interference with certain adhesive formulations, and the broader issue of favouring selective pressures that may contribute to antimicrobial resistance (19 - 21). In contrast, laser irradiation operates through a physical mechanism, producing irreversible thermal damage to microbial structures without relying on chemical agents. This characteristic eliminates the risk of chemical residues, reduces dependence on antiseptic compounds, and avoids contributing to the global challenge of antimicrobial resistance, an aspect increasingly highlighted by international health agencies (22). Although diode lasers are not currently indispensable in routine restorative workflows and the cost may limit widespread adoption, their use as an adjunctive tool warrants consideration. The combination of non-chemical antimicrobial action, absence of resistance-driving mechanisms, and potentially favourable effects on dentin adhesion makes laser-based decontamination an avenue worth exploring, particularly in minimally invasive restorative protocols that aim to reduce reliance on chemical disinfectants. Larger and more comprehensive studies, incorporating multispecies biofilms and assessments of adhesive performance, are needed to clarify the extent to which diode irradiation can enhance clinical outcomes.

## Conclusions

Within the limits of this pilot in-vitro study, 980 nm diode laser irradiation significantly reduced S. mutans contamination in standardized cavities, achieving an antimicrobial effect comparable to 0.2% chlorhexidine. Although chlorhexidine remains the most reliable and practical option in routine restorative dentistry, the laser technology demonstrated the ability to reduce bacterial load under the experimental conditions tested, without the use of chemical agents. These findings do not support changes in current clinical practice at this stage, but they provide preliminary evidence that may warrant further translational research. The absence of chemical residues, the lack of contribution to antimicrobial resistance, and the possible favourable influence on dentin substrate conditioning suggest that diode lasers could be considered as an adjunctive approach in specific restorative scenarios. Larger and clinically targeted studies are needed to determine if these "in vitro" results could be translated into significant clinical benefits.

## Figures and Tables

**Table 1:** Bacterial growth (CFU/ml) among groups.

Treatment	n	Mean (CFU/ml)	SD (CFU/ml)	Median (CFU/ml)	Min (CFU/ml)	Max (CFU/ml)
C	7	3.26×106	1.82×106	2.30×106	1.55×106	6.50×106
CHX	10	0	0	0	0	0
L	30	1.47×103	5.43×103	0	0	2.84×104

1

**Table 2:** Pairwise comparisons with Bonferroni correction.

GROUPS COMPARED	P VALUE
C vs CHX	p<0.001
C vs L	p<0.001
CHX vs L	p=0.253

2

**Table 3:** Binary Growth Analysis. Statistical test: Fisher’s exact test, Overall significance: p < 0.001.

TREATMENT	Positive samples (n)	Total samples (n)	Positivity rate (%)
C	7	7	100%
CHX	0	10	0%
L	5	30	16.7%

3
